# Endothelial Dysfunction and Hemostatic System Activation in Relation to Shift Workers, Social Jetlag, and Chronotype in Female Nurses

**DOI:** 10.3390/ijms26020482

**Published:** 2025-01-08

**Authors:** Gleb Saharov, Barbara Salti, Maram Bareya, Anat Keren-Politansky, Muhammed Fodi, Tamar Shochat, Yona Nadir

**Affiliations:** 1 Thrombosis and Hemostasis Unit, Rambam Health Care Campus, Haifa 3109601, Israelfodi.mh@gmail.com (M.F.); 2 Department of Nursing, Faculty of Social Welfare and Health Sciences, University of Haifa, Haifa 3109601, Israel; tshochat@univ.haifa.ac.il

**Keywords:** endothelial dysfunction, coagulation, shift workers, social jetlag, chronotype

## Abstract

Circadian misalignment, due to shiftwork and/or individual chronotype and/or social jetlag (SJL), quantified as the difference between internal and social timing, may contribute to cardiovascular disease. Markers of endothelial dysfunction and activation of the coagulation system may predict cardiovascular pathology. The present study aim was to investigate the effects of shift work, SJL, and chronotype on endothelial function and coagulation parameters. One hundred female nurses underwent endothelial function testing using the EndoPAT and blood sampling for coagulation markers, repeated at 06:00–9:00 and 18:00–21:00. We found that compared with day workers, endothelial function and fibrinogen levels were lower (*p* = 0.001, *p* = 0.005, respectively) and the procoagulant parameters of plasminogen activator inhibitor-1 (PAI-1) and heparanase level and activity were higher amongst shift workers (*p* = 0.009, *p* = 0.03, *p* = 0.029, respectively). High SJL was associated with lower endothelial function (*p* = 0.002) and higher PAI-1, heparanase procoagulant activity, heparanase level, and D-Dimer level (*p* = 0.004, *p* = 0.003, *p* = 0.021, *p* = 0.006, respectively). In the late chronotype, PAI-1 and heparanase procoagulant activity were higher than in the early chronotype (*p* = 0.009, *p* = 0.007, respectively). Diurnal variation was found for PAI-1, von-Willebrand factor (vWF), heparanase, and heparan-sulfate with higher levels in the mornings. The correlation between shift/day workers and SJL or chronotype was moderately strong, indicating that SJL and chronotype are independent factors. In conclusion, findings suggest endothelial impairment and increased thrombotic risk in nurses working in shifts or with high SJL or late chronotype. The thrombotic risk is increased in the morning independent of circadian misalignment cause. These findings strengthen the importance of the alliance to the biological daily rhythm in daily life. Further research is needed to evaluate inhibitors of heparanase to attenuate the thrombotic risk in individuals with circadian misalignment.

## 1. Introduction

There are increasing data supporting linkages between irregular work hours and heart and vascular morbidity. Several studies have reported that the risks of developing ischemic stroke or coronary events are higher in shift workers as compared to day workers [1,2,3]. A meta-analysis demonstrated a duration response association between shift work and increased cardiovascular disease morbidity and mortality [4]. In addition, several studies have found increased markers of endothelial dysfunction in shift workers [5,6] before the clinical presentation of cardiovascular disease. Chronotype, an individual’s natural circadian rhythm/internal timing in terms of specific sleep and wake times, may also relate to other physiological characteristics such as core body temperature, blood flow, blood pressure, and hormone secretion. Circadian misalignment refers to the discrepancy between an individual’s chronotype and social timing, with the term “social jetlag (SJL)” quantifying the discrepancy between timings of sleep on workdays and free days [7]. SJL is influenced by various factors including the extent of social and environmental mismatches, individual differences in circadian sensitivity, and the cumulative effects of such mismatches over time. Although travelers normally adjust to a new time zone within a few days, most shift workers never adjust to the SJL related to their work hours, as they are continuously exposed to the same natural daylight cycle. Late chronotype and high SJL are associated with increased risk for cardiovascular disease [8,9,10], although the mechanistic explanation of increased risk of thrombosis is poorly understood.

Heparanase, an enzyme that degrades heparan sulfate (HS) chains, is known to augment angiogenesis, metastasis, and inflammation [11,12]. We previously validated that heparanase might also affect the hemostatic system in an enzymatic-independent manner [13,14,15]. Our findings showed that heparanase directly increased tissue factor (TF) activity, resulting in enhanced factor Xa production and subsequent activation of the coagulation system [15]. Our earlier studies demonstrated that heparanase up-regulates the expression of TF [13] and interacts with the tissue factor pathway inhibitor (TFPI) on the cell surface membrane of endothelial and tumor cells, ensuing by TFPI dissociation and enhanced cell surface coagulation activity [14]. We previously demonstrated that higher levels of the heparanase, TF, TFPI, and TFPI-2 tetrad are present in hypercoagulable states such as malignancy, recurrent abortions, pregnancy, and infection [16,17], mainly in the tissue microcirculation. The issue of causal relation between heparanase and thrombosis has been addressed in the literature. Baker et al. have reported that heparanase-overexpressing mice generate a larger thrombus within a shorter period of time compared to control mice in arterial injury and stent occlusion models [18]. The main objective of the present study was to determine if there are correlations between endothelial dysfunction and specific coagulation activation parameters with shift working, SJL, and chronotype. For this aim, 100 female nurses underwent endothelial function testing using the EndoPAT and blood sampling for coagulation markers, repeated at 06:00–9:00 and 18:00–21:00. We hypothesized that shift working, high SJL, and late chronotype will be in correlation with endothelial dysfunction and activation of the coagulation system.

## 2. Results

**Demographic characteristics of shift workers and day workers**. **Recruitment flow chart was added to the supplementary material.** The demographic characteristics differed as day workers were older and had more work experience. In addition, they had more advanced positions and longer sitting hours. No statistically significant difference was found in other parameters including birth control pill use, BMI, smoking, family history of heart disease at a young age, and menstruation timing before the first blood test was drawn (Table 1).

**Endothelial dysfunction and activation of the coagulation system are higher in shift workers.** We found that evening RHI was significantly lower in shift workers as compared with day workers, implying increased endothelial dysfunction in shift workers. The difference did not reach statistical significance in the morning measurement. In accordance, PAI-1, which increases coagulation system activation, was significantly higher in the evening measurements in the shift workers as compared with day workers and reached borderline significance in the morning measurement. Heparanase levels and heparanase procoagulant activity were all higher in the shift workers as compared with day workers independent of the time of measurement. Notably, lower fibrinogen levels in shift workers may indicate a consumption coagulopathy (Table 2).

**Demographic characteristics of low SJL and high SJL.** No statistically significant difference was found in all evaluated demographic parameters between the two groups (Table 3).

**Endothelial dysfunction and activation of the coagulation system are higher in the high SJL group.** We found that morning and evening RHI were lower in the high SJL group as compared with the low SJL group, showing increased endothelial dysfunction in the first group. Morning and evening PAI-1, heparanase levels, morning heparanase procoagulant activity, and morning D-Dimer levels demonstrated increased activation of the coagulation system in the high SJL group as compared with the low SJL group (Table 4).

**Demographic characteristics of early chronotype and late chronotype**. The majority of nurses with early chronotype were older and employed in more senior positions. In addition, more smokers were in the early chronotype group. No statistically significant difference was found in other parameters including work experience, birth control pill use, BMI, family history of heart disease at a young age, and menstruation timing before the first blood test was drawn (Table 5).

**Activation of the coagulation system was higher in the late chronotype group.** We found that morning PAI-1 and evening heparanase procoagulant activity that indicate increased activation of the coagulation system were higher in late chronotypes as compared with early chronotypes. Other parameters did not significantly differ (Table 6).

**Diurnal variation in endothelial dysfunction and coagulation system parameters.** Levels of PAI-1 (*p* = 0.0001), vWF (*p* = 0.005), heparanase (*p* = 0.019), and HS (*p* = 0.005) were significantly higher in the morning as compared with the evening. Other evaluated parameters did not differ significantly (Table 7). Since heparanase, as an enzyme, degrades HS chains from the cell surface, free HS chains in the plasma reflect the heparanase level.

**Day or shift workers, chronotype, and SJL correlations.** Interestingly, the correlation between day or shift workers (designated 1 or 2, respectively) and chronotype as a linear parameter or SJL as a linear parameter was moderately strong (0.426, *p* = 0.0001 and 0.37, *p* = 0.0001, respectively). These results indicate that day workers are earlier chronotypes and have a lower SJL, while shift workers are later chronotypes and have a higher SJL. The correlation between chronotypes and SJL was also moderately strong (0.374, *p* = 0.0001), indicating that late chronotypes have higher SJL while early chronotypes have less SJL (Table 8).

## 3. Discussion

Previously, we demonstrated an increase in activation of the coagulation system in nurse shift workers as compared to day workers [19]. In the present study, we significantly increased the number of evaluated nurses. We added a dynamic evaluation of endothelial dysfunction by EndoPAT and correlated results to social jetlag and chronotype. We found that although day workers were older, heavier, and had more sitting hours, their endothelial dysfunction was lower as compared with shift workers. In addition, the procoagulant parameters of PAI-1 and heparanase level and activity were significantly higher in shift workers as compared with day workers. Lower fibrinogen levels in shift workers may indicate consumption coagulopathy. High SJL was also associated with increased endothelial dysfunction and higher PAI-1, heparanase level, heparanase procoagulant activity, and D-Dimer levels. In the late chronotype, although this group was younger, worked fewer hours, and included fewer smokers, PAI-1 and heparanase procoagulant activity were higher. These new findings support an explanation for increased thrombotic risk in shift workers, higher SJL, and late chronotype. Correlation between shift/day workers and SJL or chronotype was moderately strong, indicating that shift/day work is not the dominant parameter affecting the hemostatic status and that SJL and chronotype are independent factors.

In our study, the shift workers were 35.3 ± 7.5 years old and the day workers were 44.0 ± 4.5 years old. We reviewed the literature for evidence that fibrinogen levels differ significantly with age. Turek et al., who analyzed samples of 5840 persons aged 18–65 years, found that the mean level of fibrinogen at 30–39 years was 282, and at 40–49 years, it was 295 [20]. In our study, the morning fibrinogen level in shift workers was 227, and the level in day workers, who were older, was 252. Although the difference was higher than expected for age, we cannot exclude the possibility that the difference was attributed to the effect of aging. PAI-1 levels were higher in the shift workers even though they were younger. The fact that PAI-1 levels tend to increase with age [21] strengthens the conclusion that shift workers are at increased risk of thrombosis.

PAI-1 was demonstrated to be associated mainly with arterial thrombotic events. Tofler et al. [22] evaluated PAI-1 levels in 3203 subjects without known cardiovascular events and followed them for 10 years. The difference in PAI-1 in subjects with a thrombotic event compared with those without thrombosis was 29.1 ng/mL versus 22.1, respectively (a 32% increase in PAI-1 level). In our study, levels of PAI-1 in shift workers were higher by 32% compared with day workers; in low SJL were higher by 34% compared with high SJL; and in early chronotype were higher by 31% compared with late chronotype. Hence, these differences in the present study groups in PAI-I may indicate an increased risk of thrombosis.

Heparanase was demonstrated to activate the hemostatic system by several mechanisms [13,14,18]. Increased levels of heparanase were demonstrated in numerous hypercoagulable clinical set-ups, including women using oral contraceptives [23], women at delivery [24], patients following orthopedic surgery [25], patients with diabetic foot [26], and patients with lung cancer [27], retinal vein thrombosis [28], and prosthetic heart valve thrombosis [29]. The present study adds more data on the involvement of heparanase in the activation of the hemostatic system. As heparanase and PAI-1 are harbored in the endothelial cells, it is possible that the high levels of these two parameters that were observed resulted from endothelial injury.

Heparanase enhances not only thrombosis but also inflammation and cancer progression [30]. Although we did not evaluate specific inflammatory markers, heparanase was shown to enhance the inflammatory response by several mechanisms [31,32]. High heparanase levels in shift workers may explain their previously documented increased inflammatory markers [33,34,35]. Regarding malignancy, most published studies focus on the relationship between shift work and breast cancer development. There is some evidence from cohort studies that shift work increases the risk of developing breast cancer. There are many fewer studies that examined the correlation between shift work and other types of cancer and evidence is scarce [36]. It is possible that higher heparanase levels and procoagulant activity found in shift workers may contribute to an increase in inflammatory markers and possibly, the enhanced risk of cancer.

Increased risk of emotion dysregulation in shift workers [37] is strongly associated with chronic pro-inflammatory states or immune-related disorders [38]. Given that heparanase enhances not only thrombosis but also inflammation, emotion dysregulation and inflammation may represent a link between shift work and increased pro-thrombotic risk and might provide potential targets for interventions.

Daily diurnal variation in PAI-1, vWF, heparanase, and HS levels are intriguing results. PAI-1 was previously demonstrated to peak in the morning and to correlate with cardiovascular events [39,40]. vWF daily diurnal variation is less documented and has been evaluated only in very small-scale studies [41]. Heparanase and HS levels, as far as we know, were not previously evaluated for daily variation. The circadian pattern may point to the involvement of the endocrine system in hemostasis. Cortisol is a hormone that is expressed in the body in a circadian rhythm. In 90 percent of healthy adults, cortisol peaks within 45 min of awakening, declines throughout the day, and begins to rise during the night hours [42,43]. Collagen and fibrinogen are known to contribute to platelet adhesion and aggregation during thrombus formation. Induruwa et al. demonstrated that platelet collagen receptor Glycoprotein VI also recognizes fibrinogen and fibrin [44]. Interestingly, although the half-life of collagen molecules is months to years while that of most of the coagulation proteins is 1–2 days, diurnal variation in collagen expression was demonstrated, emphasizing the significance of diurnal expression regulation of proteins [45,46].

All participants in the study were females and there was no difference in the last menstruation date before the first blood test was drawn between the groups. Estradiol also demonstrates a circadian rhythm of morning peak, similar to cortisol [47]. Cortisol and estradiol are known to increase the risk of thrombosis. Cortisol was shown to mainly increase levels of factor VIII, vWF, and PAI-1 [48,49]. In addition, it was previously demonstrated that heparanase and HS levels are upregulated by estrogen [23,50,51]. Thus, hormonal daily variation may explain the daily changes in hemostatic parameters.

In the current study, we did not evaluate the participant’s platelets. Nakao et al. demonstrated that although platelet aggregation did not increase, there was an increase in serum thromboxane B2 in healthy medical staff after night-shift work. They concluded that this platelet hypersensitivity may be one of the mechanisms underlying the significant association between night-shift work and adverse cardiovascular outcomes [52].

One of the limitations of the study is that it was evaluated in a specific group of female nurses, albeit a very homogenous one. Studies in male nurses and other sub-groups of shift workers should be further investigated. Another limitation is the study’s cross-sectional nature, which prevents the observation of temporal effects. Longitudinal studies are needed to explore how variations in shift work schedules or chronotypes influence endothelial function and coagulation markers over time. In addition, the study does not consider psychological stress and pro-inflammatory markers, which are key factors known to influence both endothelial function and coagulation proteins.

**Figure 1:**
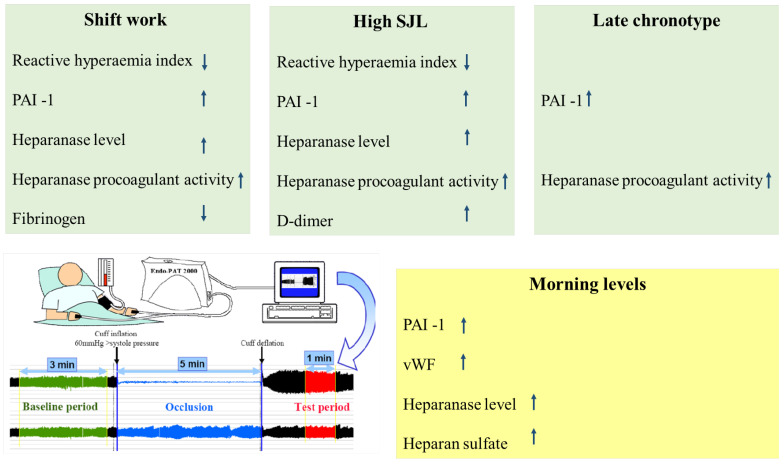
**Graphical summary.** Shift work, high social jet lag (SJL), and late chronotype in female nurses are associated with a reduced reactive hyperemia index that indicates endothelial dysfunction and increased hemostatic parameters that show activation of the coagulation system. Elevated coagulation parameters in the morning as compared with evening support daily diurnal variation in hemostatic proteins. A cartoon of the EndoPAT system measurement of endothelial dysfunction is shown (lower panel, left). Abbreviations: plasminogen activator inhibitor-1 (PAI-1), von Willebrand factor (vWF).

In conclusion, the present study demonstrated evidence of endothelial dysfunction in shift workers and women with high SJL in female nurses. Coagulation markers indicating activation of the coagulation system were significantly higher in shift workers, high SJL, and late chronotype groups. The thrombotic risk was increased in the morning independent of the circadian misalignment cause, as summarized in Figure 1. These findings strengthen the importance of the alliance in the daily life of an individual to the biological circadian rhythm. Misalliance may contribute to morbidity. Further research is needed to evaluate inhibitors of heparanase to attenuate the risk of thrombosis in patients with circadian rhythm misalliance.

## 4. Material and Methods

### 4.1. Study Group

The study was approved by the Institutional Review Board of Rambam Health Care Campus Rambam (No. 0081-20-RMB). An email to all nurses in the hospital that invited female nurses to participate in the research was distributed. After obtaining written informed consent, 100 female nurses were recruited. The inclusion criteria were age 25–50 years, body mass index (BMI) ≤ 30, and working at least 75% in either regular day work or rotating day–evening–night shifts at Rambam. None of the day workers were occasionally doing also shifts. The exclusion criteria were the presence of diabetes, hypertension, pregnancy, and use of any chronic medications (excluding hormonal replacement treatments). All participants were Caucasians. In order to reduce the number of confounding factors, the tests were conducted in the morning and evening of the same day. The tests were performed only when the nurse was on a morning shift, not during an evening shift or following a night shift.

Chronotype was estimated by self-reporting using the Munich Chrono Type Questionnaire (MCTQ) for day workers and MCTQ^shift^ adjusted for shift workers [53]. The early chronotype was defined as mid-sleep (midpoint between sleep onset and wake-up) on free days (MSF) < 4 AM and the late chronotype was defined as MSF ≥ 4 AM according to the previous publication [54]. SJL was defined as circadian misalignment, evaluated as the difference between the sleep time on workdays and free days over a 2-week period [54]. SJL was defined as <1.2 h (low SJL) or ≥1.2 h (high SJL), thus dividing the number of participants at the median.

### 4.2. Reagents and Antibodies

A single-chain GS3 heparanase gene construct, comprising the 8 and 50 kDa heparanase subunits (8 + 50), was purified from a conditioned medium of baculovirus-infected cells. GS3 heparanase was assayed for the presence of bacterial endotoxin (Biological Industries, Beit Haemek, Israel) using the gel–clot technique (limulus amebocyte lysate—LAL test) and found to contain < 10 pg/mL of endotoxin [13].

### 4.3. Endothelial Function Assessment

To assess endothelial status, we performed noninvasive endothelial function tests with the EndoPAT system (Itamar Medical Ltd., Caesarea, Israel), a US Food and Drug Administration (FDA)-cleared non-invasive test to evaluate the level of endothelial function. EndoPAT assesses digital flow-mediated dilation during reactive hyperemia using measurements from both arms—the occluded side that is applied by near-diastolic external pressure and the control side [55,56]. EndoPAT provides an endothelial function Reactive Hyperemia Index (RHI) that is the post-to-pre occlusion signal ratio in the occluded side, normalized to the control side. Reactive hyperemia is a well-established technique for noninvasive assessment of peripheral microvascular function and a predictor of all-cause cardiovascular morbidity and mortality [57]. Measurements were conducted in accordance with conditions specified by the manufacturer. All tests were performed in the supine position in quiet surroundings, on a comfortable bed in a dimly lit room, with a temperature of 21–24 °C. Pneumatic probes were placed on each index finger and a blood pressure cuff on one arm. With the arms at rest on comfortable arm supports, probes were inflated to a subdiastolic pressure to avoid distal venous pooling, thereby inhibiting the veno-arteriolar vasoconstriction reflex. The recording was initiated after 25 min of rest. After 5 min baseline recording, the blood pressure cuff was inflated to 60 mmHg above systolic blood pressure and no less than 200 mmHg. Occlusion was confirmed by visual confirmation of complete attenuation of the PAT signal from the test arm. After 5 min occlusion, the cuff was deflated and the recording continued for 5 min during the reactive hyperemia phase. Recordings from the non-occluded arm served as an internal control correcting for systemic changes in vascular tone. RHI was calculated as the index of signal amplitude pre-to-post occlusion in the occluded arm, divided by the same ratio in the control arm [56]. The low index indicates an endothelial dysfunction. All participants underwent the EndoPAT test at 06:00–9:00 and 18:00–21:00 on the same day in the Thrombosis and Hemostasis Unit at Rambam by a qualified nurse.

### 4.4. Blood Coagulation Parameters

Ten milliliters of blood were drawn to 3.2% citrated tubes at 06:00–9:00 and 18:00–21:00, before the EndoPAT assessment. Plasma was obtained by centrifugation (1500× *g* for 15 min at room temperature). Fibrinogen was performed on fresh plasma samples. All other coagulation assays were performed on the thawed frozen plasma samples. Plasma samples were frozen after a second centrifugation at 2000× *g* for 5 min in aliquots, at −70 ± 5 °C. Prior to testing, plasma aliquots were thawed in a 37 ± 0.5 °C water bath for 15 min, after which a von Willebrand factor (vWF) assay was performed on the STA-R Evolution analyzer (Diagnostica Stago, Gennevilliers, France). STALiatest^®^ D-DI or STA-Liatest^®^ vWF:ag kits were employed for D-dimer and vWF. The total PAI-1 level was measured using an enzyme-linked immunosorbent assay (ELISA) with Asserachrom^®^ PAI-1 (Diagnostica Stago, Gennevilliers, France).

### 4.5. Heparanase Procoagulant Activity Assay

A basic experiment of factor Xa generation was performed in the following manner, with given concentrations as the final ones. We incubated 25 µL of the plasma, recombinant human factor VII (0.04 μM), and plasma-derived human factor X (1.4 μM) in a 50 μL assay buffer [0.06 M Tris, 0.04 M NaCl, 2 mM CaCl_2_, 0.04% *w*/*v* bovine serum albumin, pH 8.4] to a total volume of 125 μL in a 96-well sterile plate. After 15 min at 37 °C, the chromogenic substrate to factor Xa was added (1 mM). After 20 min, the reaction was stopped with 50 μL of glacial acetic acid and the level of Xa generation was determined using an ELISA plate reader (PowerWave XS; BioTek, Winooski, VT, USA). Heparins were shown to abrogate the TF/heparanase complex [14]. In parallel, the same assay was performed except that fondaparinux (2.5 μg/mL) was added to the assay buffer. Bovine factor Xa diluted in the assay buffer was used to generate a standard curve. The subtraction of the first assay result from the second assay result determined the Heparanase procoagulant activity [24].

### 4.6. Heparanase ELISA

Wells of microtiter plates were coated (18 h, 4 °C) with 2 μg/mL of anti-Heparanase monoclonal antibody 4B11 in 50 μL coating buffer (0.05 M Na_2_CO_3_, 0.05 M NaHCO_3_, pH 9.6). The plate was covered with adhesive plastic and incubated overnight at 4 °C. The next day, wells were blocked with 2% BSA in PBS for 1 h at room temperature. Diluted samples (100 μL) were loaded in duplicates and incubated for 2 h at room temperature, followed by the addition of 100 μL polyclonal antibody 63 IgG (1 μL/mL) for an additional period of 2 h at room temperature. HRP-conjugated goat anti-rabbit IgG (1:20,000) in blocking buffer was added (1 h, room temperature) and the reaction was visualized by the addition of 100 μL chromogenic substrate (TMB; MP Biomedicals, Berlin, Germany) for 15 min. The reaction was stopped with 100 μL H_2_SO_4_ and absorbance at 450 nm was measured using the ELISA plate reader. Plates were washed four times with washing buffer (PBS, pH 7.4 containing 0.1% (*v*/*v*) Tween 20) after each step. As a reference for quantification, a standard curve was established by serial dilutions of recombinant 8 + 50 GS3 Heparanase (390 pg/mL-25 ng/mL) [58].

### 4.7. Heparan Sulfate Chromogenic Assay

Levels of HS were measured in the following way: 16 mg of dimethylmethylene blue (DMMB, Sigma-Aldrich, Rehovot, Israel) were dissolved in 1 L of double distilled water containing 3.04 g glycine, 1.6 g NaCl, and 95 mL of 0.1 M acetic acid. The solution was filtered using Whattman 3 MM. A standard curve was prepared using HS derived from a bovine kidney (H7640, Sigma-Aldrich, Rehovot, Israel). To 20 μL of a sample, 5 μL of protamine sulfate (10 mg/mL, Fresenius Kabi, Toronto, Canada) was added followed by the addition of 20 μL of a sample to 200 μL of DMMB solution. The absorbance was immediately read using a plate reader at 525 nm. Each sample was evaluated with and without protamine sulfate, after which the subtraction of the first assay result from the second assay result determined the HS level in a sample [59].

### 4.8. Statistical Analysis

Data were evaluated using SPSS software for Windows, version 13.0 (SPSS Inc., Chicago, IL, USA). Statistics were calculated by one-way ANOVA in order to compare endothelial and coagulation parameters, independent of age and BMI. A paired *t*-test was used to compare diurnal variation in these parameters. Pearson’s correlation test was used to compare shift work, SJL, and chronotype. Values were reported as mean ± SD. The significance level was set at *p* < 0.05.

## Figures and Tables

**Table 1:** Demographic characteristics of the day workers versus shift workers.

	Day Workers n = 49	Shift Workers n = 51	*p*-Value
Age (years)	44.0 ± 4.5	35.3 ± 7.5	**0.001**
Total nurse experience (years)	19.9 ± 6.4	10.3 ± 6.7	**0.001**
Experience in current work (years)	7.5 ± 5.9	8.3 ± 6.4	0.52
Scope of the job			**0.024**
100%	41 (84%)	30 (59%)	
88%	3 (6%)	8 (16%)	
75%	5 (10%)	13 (25%)	
Birth control pills use (n)	6 (12%)	5 (10%)	0.76
BMI	24.2 ± 3.6	23.7 ± 3.5	0.49
Smoking (n)	8 (16%)	5 (10%)	0.38
Family history of heart disease at young age (n)	10 (20%)	8 (16%)	0.54
Last menstruation date before the first blood test			0.64
no menstruation	8 (16%)	4 (8%)	
1–10 days	13 (27%)	15 (29%)	
11–20 days	9 (18%)	10 (20%)	
21–35 days	19 (39%)	22 (43%)	
Hours of sitting during working day			**0.001**
0–1	4 (8%)	22 (43%)	
1–3	30 (61%)	25 (49%)	
3–6	15 (31%)	4 (8%)	

Abbreviations: body mass index (BMI). Bold values are values that reached significance of *p* < 0.05.

**Table 2:** Endothelial dysfunction and activation of the coagulation system in day workers and shift workers.

	Day Workers n = 49	Shift Workers n = 51	*p* Value
Morning reactive hyperaemia index	0.67 ± 0.26	0.58 ± 0.27	0.083
Evening reactive hyperaemia index	0.71 ± 0.21	0.62 ± 0.3	**0.001**
Morning plasmin activator inhibitor-1 (ng/mL)	13 ± 4.83	15.9 ± 9.1	0.053
Evening plasmin activator inhibitor-1	8.3 ± 3.48	11.1 ± 6.48	**0.009**
Morning von Willebrand factor (%)	136 ± 42	126 ± 43	0.28
Evening von Willebrand factor	127 ± 41	117 ± 44	0.25
Morning heparanase levels (pg/mL)	2653 ± 1247	3258 ± 1516	**0.03**
Evening heparanase levels	2543 ± 1122	3121 ± 1513	**0.03**
Morning heparanase procoagulant activity (ng/mL)	414 ± 279	585 ± 513	**0.043**
Evening heparanase procoagulant activity	341 ± 259	555 ± 626	**0.029**
Morning D-Dimer (ng/mL)	294 ± 152	337 ± 195	0.22
Evening D-Dimer	312 ± 235	369 ± 241	0.23
Morning fibrinogen (mg/dL)	252 ± 47	227 ± 38	**0.005**
Evening fibrinogen	247 ± 46	228 ± 41	**0.033**
Morning heparan sulfate levels (µg/mL)	27 ± 25	34 ± 32	0.3
Evening heparan sulfate levels	18 ± 16	26 ± 24	0.28

Results were adjusted to age and BMI.

**Table 3:** Demographic characteristics of low social jetlag versus high social jetlag.

	Low SJL < 1.2 h n = 50	High SJL ≥ 1.2 h n = 50	*p*-Value
Age (years)	40.9 ± 6.6	35.4 ± 8.3	0.099
Total nursing experience (years)	16.4 ± 7.5	13.7 ± 8.6	0.098
Experience in current work (years)	7.6 ± 5.8	8.1 ± 6.5	0.68
Scope of the job			0.62
100%	36 (74%)	35 (68%)	
88%	6 (12%)	5 (10%)	
75%	7 (14%)	11 (22%)	
Birth control pills use (n)	3 (6.1%)	8 (15.7%)	0.20
BMI	23.7 ± 3.3	24.2 ± 3.8	0.48
Smoking (n)	8 (16.3%)	5 (9.8%)	0.38
Family history of heart disease at young age (n)	9 (18.4%)	9 (17.6%)	0.93
Last menstruation date before the first blood test			0.69
No menstruation	6 (12%)	6 (12%)	
1–10 days	15 (31%)	13 (25.5%)	
11–20 days	7 (14%)	12 (23.5%)	
21–35 days	21 (43%)	20 (39%)	
Hours of sitting during working day			0.23
0–1	9 (18%)	17 (33%)	
1–3	30 (61%)	25 (49%)	
3–6	10 (20%)	9 (18%)	

Abbreviations: body mass index (BMI).

**Table 4:** Endothelial dysfunction and activation of the coagulation system in the low SJL and high SJL groups.

	Low SJL < 1.2 h n = 50	High SJL ≥ 1.2 h n = 50	*p* Value
Morning reactive hyperaemia index	0.69 ± 0.28	0.56 ± 0.23	**0.014**
Evening reactive hyperaemia index	0.7 ± 0.23	0.53 ± 0.28	**0.002**
Morning plasmin activator inhibitor-1 (ng/mL)	12.3 ± 5.6	16.5 ± 8.4	**0.004**
Evening plasmin activator inhibitor-1	8.8 ± 5.1	10.5 ± 5.4	0.12
Morning von Willebrand factor (%)	124 ± 43	136 ± 42	0.16
Evening von Willebrand factor	118 ± 38	125 ± 46	0.46
Morning heparanase levels (pg/mL)	2539 ± 995	3366 ± 1637	**0.003**
Evening heparanase levels	2548 ± 1067	3115 ± 1553	**0.037**
Morning heparanase procoagulant activity (ng/mL)	401 ± 358	596 ± 459	**0.021**
Evening heparanase procoagulant activity	442 ± 439	458 ± 542	0.87
Morning D-Dimer (ng/mL)	267 ± 141	365 ± 194	**0.006**
Evening D-Dimer	311 ± 245	370 ± 230	0.22
Morning fibrinogen (mg/dL)	238 ± 47	241 ± 42	0.74
Evening fibrinogen	242 ± 41	234 ± 48	0.35
Morning heparan sulfate levels (µg/mL)	31 ± 30	30 ± 28	0.9
Evening heparan sulfate levels	25 ± 21	24 ± 22	0.8

Results were adjusted to age and BMI.

**Table 5:** Demographic characteristics of early chronotype versus late chronotype.

	Early Chronotype MSF < 4 AM n = 57	Late Chronotype MSF ≥ 4 AM n = 43	*p*-Value
Age (years)	40.86 ± 6.7	37.9 ± 8.4	**0.051**
Total nurse experience (years)	16.3 ± 7.8	13.3 ± 8.3	0.068
Experience in current work (years)	7.6 ± 5.5	8.3 ± 6.9	0.56
Scope of the job			**0.012**
100%	46 (81%)	25 (58%)	
88%	2 (3%)	9 (21%)	
75%	9 (16%)	9 (21%)	
Birth control pills use (n)	6 (10.5%)	5 (11.6%)	0.86
BMI	23.8 ± 3.3	24.2 ± 3.8	0.58
Smoking (n)	11 (19%)	2 (4.7%)	**0.037**
Family history of heart disease at young age (n)	12 (21%)	6 (14%)	0.36
Last menstruation date before the first blood test			0.78
no menstruation	8 (14%)	4 (9%)	
1–10 days	15 (26%)	13 (30%)	
11–20 days	12 (21%)	7 (16%)	
21–35 days	22 (39%)	19 (44%)	
Hours of sitting during working day			0.11
0–1	11 (19%)	15 (35%)	
1–3	32 (56%)	23 (53%)	
3–6	14 (25%)	5 (12%)	

Abbreviations: body mass index (BMI).

**Table 6:** Endothelial dysfunction and activation of the coagulation system in early chronotype and late chronotype groups.

	Early Chronotype MSF < 4 AM n = 51	Late Chronotype MSF ≥ 4 AM n = 49	*p* Value
Morning reactive hyperaemia index	0.65 ± 0.28	0.59 ± 0.23	0.27
Evening reactive hyperaemia index	0.62 ± 0.23	0.61 ± 0.32	0.82
Morning plasmin activator inhibitor-1 (ng/mL)	12.7 ± 5.6	16.6 ± 8.9	**0.009**
Evening plasmin activator inhibitor-1	9.2 ± 4.2	10.4 ± 6.6	0.25
Morning von Willebrand factor (%)	126 ± 42	136 ± 43	0.28
Evening von Willebrand factor	118 ± 37	126 ± 48	0.33
Morning heparanase levels (pg/mL)	2828 ± 1327	3137 ± 1525	0.28
Evening heparanase levels	2718 ± 1303	2996 ± 1432	0.31
Morning heparanase procoagulant activity (ng/mL)	436 ± 366	586 ± 478	0.07
Evening heparanase procoagulant activity	336 ± 263	601 ± 661	**0.007**
Morning D-Dimer (ng/mL)	311 ± 142	321 ± 214	0.78
Evening D-Dimer	338 ± 238	344 ± 242	0.91
Morning fibrinogen (mg/dL)	245 ± 42	235 ± 47	0.38
Evening fibrinogen	243 ± 40	232 ± 49	0.22
Morning heparan sulfate levels (µg/mL)	31 ± 30	30 ± 29	0.9
Evening heparan sulfate levels	25 ± 23	20 ± 19	0.5

Results were adjusted to age and BMI.

**Table 7:** Diurnal variation in endothelial dysfunction and coagulation system parameters.

	Morning n = 100	Evening n = 100	*p* Value
Reactive hyperaemia index	0.62 ± 0.26	0.61 ± 0.27	0.8
Plasmin activator inhibitor-1 (ng/mL)	14.4 ± 7.4	9.7 ± 5.3	**0.0001**
von Willebrand factor (%)	131 ± 43	121 ± 42	**0.005**
Heparanase levels (pg/mL)	2961 ± 1416	2838 ± 1360	**0.019**
Heparanase procoagulant activity (ng/mL)	500 ± 422	450 ± 492	0.26
D-Dimer (ng/mL)	316 ± 176	341 ± 238	0.16
Fibrinogen (mg/dL)	239 ± 44	238 ± 45	0.6
Heparan sulfate levels (µg/mL)	31 ± 28	23 ± 19	**0.005**

**Table 8:** Shift workers, chronotype, and SJL correlations.

	Pearson’s Correlation	*p* Value
Shift/day workers/chronotype *	0.427	0.0001
Shift/day workers/SJL *	0.37	0.0001
Chronotype */SJL *	0.374	0.0001

* The parameter was evaluated as a linear parameter.

## Data Availability

Data are contained within the article.

## Supplementary Materials

The following supporting information can be downloaded at: https://www.mdpi.com/article/10.3390/ijms26020482/s1.
