# Supplementary figures and images for: Endothelial Dysfunction and Hemostatic System Activation in Relation to Shift Workers, Social Jetlag, and Chronotype in Female Nurses
Source: Int J Mol Sci. 2025 Jan 8;26(2):482. doi: 10.3390/ijms26020482 (PMC11764714; doi:10.3390/ijms26020482)

## Recruitment flow chart

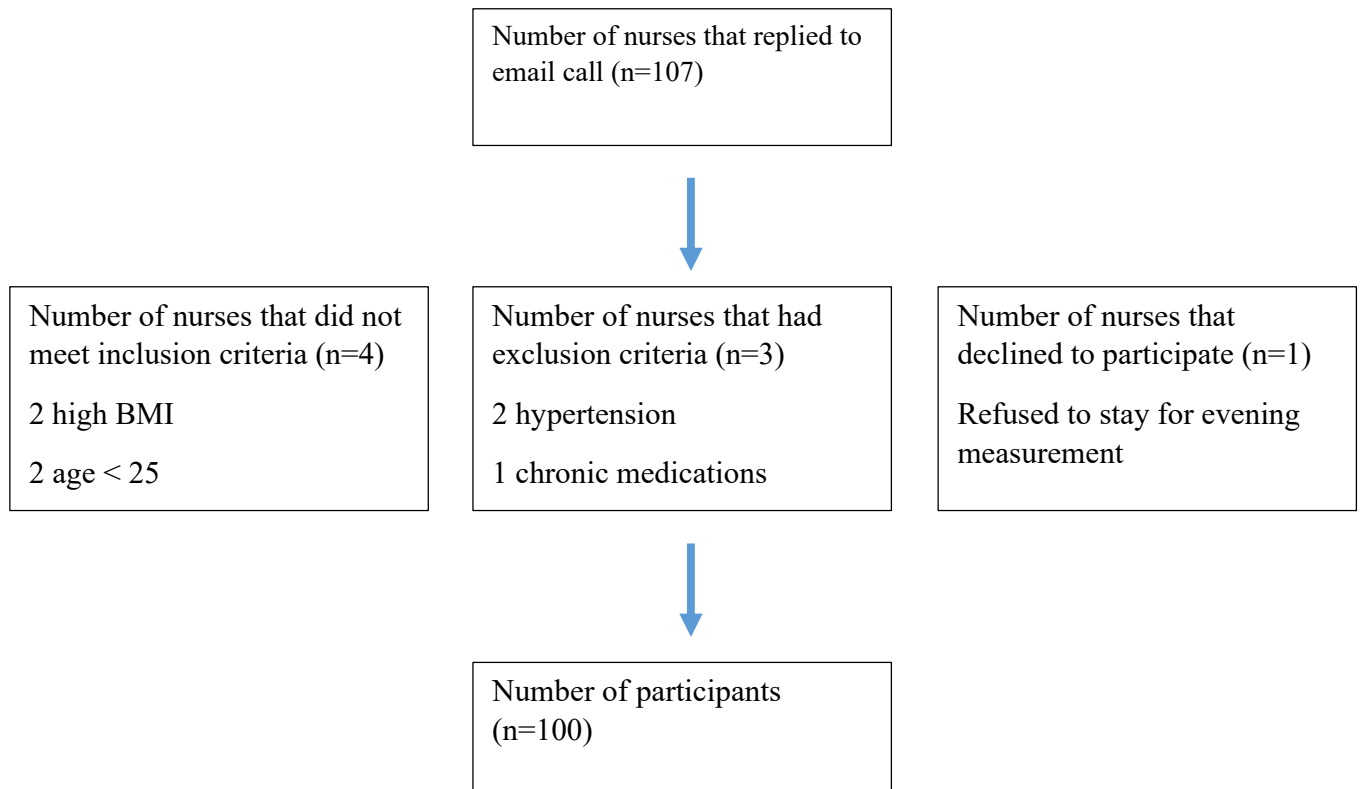

Supplement: Supplementary file 1 [file ijms-26-00482-s001.zip › ijms-3380578-supplementary.pdf]
